# Metal-Semiconductor AsSb-Al_0.6_Ga_0.4_As_0.97_Sb_0.03_ Metamaterial

**DOI:** 10.3390/ma15217597

**Published:** 2022-10-28

**Authors:** Nikolay Bert, Vitaliy Ushanov, Leonid Snigirev, Demid Kirilenko, Vladimir Ulin, Maria Yagovkina, Valeriy Preobrazhenskii, Mikhail Putyato, Boris Semyagin, Igor Kasatkin, Vladimir Chaldyshev

**Affiliations:** 1 Ioffe Institute, 26 Politekhnicheskaya Str., 194021 Saint Petersburg, Russia; 2 Rzhanov Institute of Semiconductor Physics of the Siberian Branch of the RAS, 13 Lavrentyev Prospekt, 630090 Novosibirsk, Russia; 3 Saint Petersburg State University, 7–9 Universitetskaya Nab., 199034 Saint Petersburg, Russia

**Keywords:** metal-semiconductor composite, low-temperature MBE, microstructure, AsSb nanoparticles, plasmon resonance

## Abstract

AlGaAsSb and AlGaAs films as thick as 1 μm with Al content as high as 60% were successfully grown by low-temperature (200 °C) MBE. To overcome the well-known problem of growth disruption due to a high aluminum content and a low growth temperature, we applied intermittent growth with the temperature elevation to smooth out the emerging roughness of the growth front. Post-growth annealing of the obtained material allowed us to form a developed system of As or AsSb nanoinclusions, which occupy 0.3–0.6% of the material volume. While the As nanoinclusions are optically inactive, the AsSb nanoinclusions provide a strong optical absorption near the band edge of the semiconductor matrix due to the Fröhlich plasmon resonance. Owing to the wider bandgap of the grown Al_0.6_Ga_0.4_As_0.97_Sb_0.03_ compound, we have expanded the spectral range available for studying the Fröhlich plasmon resonance. The grown metamaterial represents an optically active medium of which the formation process is completely compatible with the epitaxial growth technology of semiconductors.

## 1. Introduction

Interaction of light with metallic nanoparticles can provide localization and enhancement of the optical fields at the sub-wavelength scale. These phenomena are caused by the interaction of the light wave with the intrinsic localized excitations of the electron plasma in the nanoparticles. If an array of nanoparticles is formed in a dielectric or semiconducting medium, the dielectric and optical properties of the medium can be substantially modified. Such metal-dielectric (metal-semiconductor) composite metamaterials support Fröhlich plasmon resonances, the parameters of which depend on the properties of both the ensemble of nanoparticles and the matrix [1]. Such media can exhibit unusual linear and non-linear optical properties. Colored glasses are beautiful examples of such a linear optical response. Saturable optical absorbers operating near the localized surface plasmon resonance are modern examples of non-linear optical media in demand [2]. A system of metal nanoparticles provides much better efficiency of light-matter interaction and much shorter relaxation time after optical excitation, as compared, for instance, with a similar system of quantum dots.

It is promising to integrate the metal-semiconductor metamaterial with semiconductor lasers, light-emitting devices, and other optoelectronic components. Unfortunately, such integration is impossible for the common plasmonic materials—gold and silver, since the fabrication technology of the Au and Ag nanoparticles is not compatible with the epitaxial growth technology of III–V semiconductor compounds widely used for optoelectronics. In this case, the system of plasmonic nanoparticles can only be produced on the surface of the semiconductor nanostructure by post-growth deposition and treatments [3,4].

A unique possibility to produce an array of plasmonic nanoparticles in epitaxial layers of GaAs and AlGaAs is provided by low temperature (LT) molecular beam epitaxy (MBE) under the condition of excess As [5,6]. Thus-grown epitaxial layers of LT-GaAs exhibit a high crystalline quality but contain a high concentration of over-stoichiometric arsenic, mostly as antisite substitutes As_Ga_ [7,8]. The concentration of these defects can strongly exceed the equilibrium value and reach 2 at.%. The post-growth heat treatment at a high temperature activates the diffusion processes and leads to the self-organization of arsenic nanoinclusions in the metastable medium [6,9]. During this process, the crystalline quality of the semiconductor matrix remains high, and the material meets the standard requirements for common semiconductor heterostructures.

The presence of the array of arsenic nanoparticles does not cause any substantial changes in the optical properties of the medium within the transparency window of the GaAs matrix in the near-infrared range [6,10]. However, in the case of LT MBE of GaAs_1−*y*_Sb*_y_* and Al*_x_*Ga_1−*x*_As_1−*y*_Sb*_y_* (*x* ≈ 0.3, *y* ≈ 0.03) solid solutions, a specific optical absorption was revealed, which was attributed to the system of the AsSb nanoinclusions [11,12,13]. The absorption coefficient increased with increasing photon energy till the edge of the band-to-band transitions. This absorption was interpreted as a Fröhlich resonance in the system of plasmonic nanoparticles. In order to reliably determine the parameters of this resonance, the optical transparency window of the semiconductor Al*_x_*Ga_1−*x*_As_1−*y*_Sb*_y_* matrix should be expanded. It can be done by an increase in the aluminum concentration *x*. With this, the formation of a developed system of AsSb nanoinclusions should be provided in such a matrix. This task is not trivial since the alloys with a high aluminum concentration are much less favorable for the required self-organization of the nanoparticles [14,15].

In this paper, we show the possibility to form developed arrays of AsSb nanoparticles in the bulk of the epitaxial films of Al*_x_*Ga_1−*x*_As_1−*y*_Sb*_y_* semiconductor alloy with high aluminum concentration *x* = 60% and relatively small antimony content *y* = 3%. We determine the structure of such a composite metal-semiconductor metamaterial. We investigate the optical properties within the transparency window (λ > 600 nm) and reveal a strong plasmon-related optical absorption in a photon energy range near the Al*_x_*Ga_1−*x*_As_1−*y*_Sb*_y_* band gap.

## 2. Materials and Methods

### 2.1. Sample Growth

To consistently trace the effect of the aluminum and antimony content on the growth, structural and optical properties of the resulting material, we grew a series of four samples. The samples were grown by MBE on semi-insulating GaAs substrates with (001) ± 0.5° orientation. The LT-layers of Al*_x_*Ga_1−*x*_As and Al*_x_*Ga_1−*x*_As_1−*y*_Sb*_y_* were deposited at a substrate temperature of 200 °C. The presence of Al is known to hinder epitaxial growth due to the much lower surface mobility of Al adatoms compared to Ga ones, which leads to developing growth surface roughness. To achieve approximately similar conditions for the growth of Al-free material, the samples of LT-GaAs and LT-GaAs_1-y_Sb_y_ were grown at the temperature lowered to 150 °C.

Nominal concentrations of Al and Sb were *x* = 60% and *y* = 3%. The growth development was monitored using reflection high-energy electron diffraction (RHEED).

Before the growth, the substrate was initially heated to a temperature of 580 °C in order to remove the protecting oxide. The desorption of the oxide was monitored by RHEED through the transformation of the diffuse scattering pattern to the diffraction streaks appearance. The substrate temperature was controlled with a thermocouple mounted at the substrate holder. The temperature readings were calibrated by known temperatures of the GaAs surface reconstructions. The flux of As_4_ was *P*_As4_ = 1 × 10^15^ cm^−2^s^−1^.

At first, we grew a 0.2-μm-thick GaAs buffer layer at a substrate temperature of 580 °C with a growth rate of 1 μm/h. The growth conditions provided the (3 × 1) surface reconstruction, which led to the formation of a system of equidistant monoatomic steps on the growth surface. The terrace width was 300 ÷ 350 nm as defined by the misorientation from the singular (001) surface. When the growth of the buffer layer was completed, the gallium flux was interrupted, and the temperature was lowered. Then, epitaxial layers of either LT-GaAs, or LT-GaAs_0.97_Sb_0.03_, or LT-Al_0.6_Ga_0.4_As, or LT-Al_0.6_Ga_0.4_As_0.97_Sb_0.03_ were grown with a thickness of about 1 μm.

At the initial stage of the LT MBE, the RHEED revealed a well-developed diffraction pattern indicating a high crystalline quality of the atomically smooth growth surface. Gradually increasing with the layer thickness, a diffuse scattering appeared in the RHEED pattern, pointing to a growth surface roughening due to the mechanical stress originating from the lattice mismatch between the layer and the substrate.

To prevent a possible disruption of the epitaxial growth, which was alarmed by monitoring the RHEED pattern, we interrupted the growth and initiated an intermediate annealing. There was a single intermediate anneal for the LT-GaAs sample, and there were two anneals for the LT-GaAs_0.97_Sb_0.03_ sample performed at a temperature of 250 °C. For the LT-Al_0.6_Ga_0.4_As and Al_0.6_Ga_0.4_As_0.97_Sb_0.03_ samples, the anneals were performed every 0.16 μm and 0.10 μm, correspondingly, at a temperature of 400 °C. All the epitaxial structures were finalized by the cap layers of AlAs and GaAs as thick as 5 nm each. We did not observe any RHEED features, which could indicate substantial relaxation of the epitaxial structures.

After the growth, each wafer was cut into several parts. One of them was kept as-grown. The others were annealed at different temperatures in the range of 400–800 °C for 15 min in the MBE setup under the As_4_ overpressure.

### 2.2. Characterization Techniques

The chemical composition of the samples and the concentration of the excess arsenic incorporated during the LT MBE were evaluated by using two independent techniques—X-ray diffraction (XRD) and optical transmission and reflection.

The X-Ray diffraction was studied at XRD Research Center, SPbSU, with a Bruker D8 Discover high-resolution diffractometer equipped with a Cu-sealed tube X-Ray source (λ = 1.54056 Å) and a 4-bounce Montel monochromator. Diffraction curves were recorded around the 004 reflection of the GaAs substrate and analyzed by simulations with Leptos software (Bruker). The starting model was based on the original design of the heterostructures with the intermediate anneals taken into account. The concentration of antisite defects, As_Ga_, was estimated from the shifts of the reflections (around the 004 peak position), which resulted from the post-growth high-temperature anneals [16,17,18].

Optical reflection, transmission, and extinction investigations were carried out at room temperature with normal light incidence in wavelength ranges of 400–1000 nm and 900–1600 nm using OceanOptics QE65Pro and NIRQuest-512 spectrometers, respectively. An Osram HLX 100W 6.6A lamp with a collimator served as a source of light radiation. The spectra were recorded using the OceanOptics SpectraSuite software.

In order to investigate the light extinction coefficient behavior in the optical transparency window of the LT-Al_0.6_Ga_0.4_As_0.97_Sb_0.03_ epilayer, the GaAs substrate of the sample had to be removed. For that, the epitaxial surface of the sample was glued to an optical glass, and the substrate was dissolved by chemical etching. The transmission and reflection optical spectra were measured simultaneously at the same point on the sample surface, which made it possible to reduce the effect of Fabry–Perot interference on the extinction spectra.

The excess As concentrations in non-stoichiometric materials were determined by measurements of the optical extinction in the as-grown portion of the sample. This extinction was previously calibrated at the wavelengths of 1.0 and 1.06 µm at room temperature [19], which allowed us to calculate the concentrations of neutral antisite defects, As_Ga_, from the optical absorption coefficients.

Transmission electron microscopy (TEM) was utilized to study the microstructure of the samples including the formation of nanoparticles in the AlGaAs matrix and evaluation of their size and concentration. Electron-transparent specimens for TEM were prepared in (110) cross-sections according to the conventional procedure with preliminary thinning by mechanical processing and finishing ion sputtering. The studies were performed using a JEM-2100F electron microscope (JEOL, Tokyo, Japan) with an accelerating voltage of 200 kV equipped with an energy-dispersive X-ray spectrometer (EDXS) Quantax 400 STEM XFlash 6T 30 (Bruker AXS, Karlsruhe, Germany). Conventional TEM was exploited in both imaging and selected area electron diffraction (SAED) modes, as well as scanning transmission electron microscopy, was utilized with an annular dark-field detector (STEM-HAADF). Investigations were performed using TEM equipment owned by the Federal Joint Research Center “Materials science and characterization in advanced technology” at Ioffe Institute (Saint Petersburg, Russia).

## 3. Results

### 3.1. X-ray Diffraction

**Figure 1:**
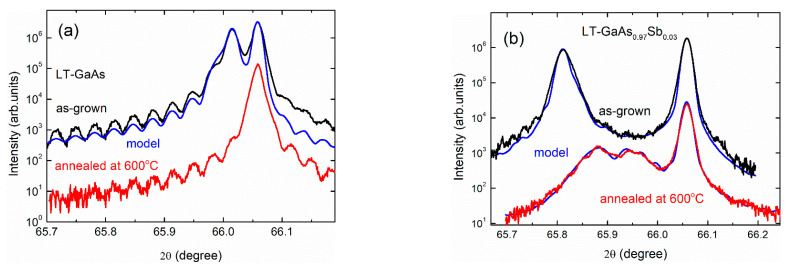

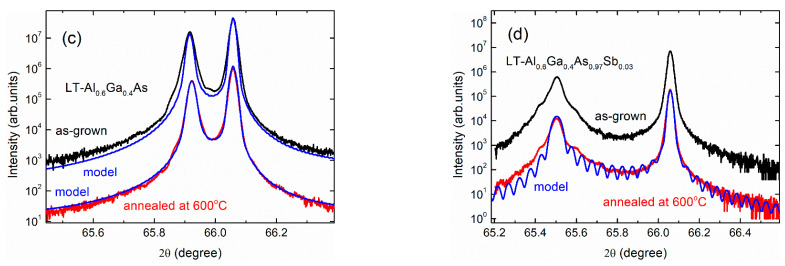
Experimental (black and red curves) and simulated (blue curves) XRD profiles around the 004 reflection of the GaAs substrate with (**a**) LT-GaAs, (**b**) LT-GaAs_0.97_Sb_0.03_, (**c**) LT-Al_0.6_Ga_0.4_As and (**d**) LT-Al_0.6_Ga_0.4_As_0.97_Sb_0.03_ layers before (black curves) and after (red curves) annealing at 600 °C. The curves related to the as-grown sample are shifted up for clarity.

Figure 1 shows the XRD profiles of the samples recorded in their as-grown state and after annealing at 600 °C. Two separate reflection peaks and thickness fringes are clearly seen on the curves for all the as-grown samples. The left peak in the XRD profiles is attributed to the 004 reflection from the epitaxial layer and the right peak originates from the substrate. By simulating the XRD profiles (blue lines in Figure 1 are the calculated curves) the values of the lattice constant *c* along the growth direction are refined.

The annealing at 600 °C causes a shift of the LT-layer-related XRD peak to larger angles. It indicates a decrease in the lattice parameter due to precipitation of the excess As primarily dissolved as antisite defects As_Ga_ in the low-temperature-grown films [6,7,8]. In Ref. [7] the corresponding change in the lattice parameter was documented as

(1) $$\frac{\Delta c}{c}=1.24\times 10^{-23}\left[{\mathrm{As}}_{\mathrm{Ga}}\right],$$

where [As_Ga_] is the concentration of the anti-site defects in the unit of cm^−3^. We use this calibration to determine the [As_Ga_] in LT-GaAs and LT-GaAs_1−*y*_Sb*_y_* samples. The values thus obtained turned out to be 4.8 × 10^19^ cm^−3^ and 7.5 × 10^19^ cm^−3^ correspondingly. It should be noted that the excess arsenic is the only reason for the lattice expansion in LT-GaAs. After annealing at 600 °C for 15 min most of the anti-site defects agglomerate into nanoinclusions. As a result, the GaAs matrix becomes nearly stoichiometric and the film-related XRD peak almost coincides with the substrate-related one, so that only a single peak appears in the experimental profile, Figure 1a.

For the epitaxial LT-GaAs_1−*y*_Sb*_y_* film, an additional lattice expansion is provided by As-Sb substitution in the anion sublattice. This expansion persists after annealing. In the experimental XRD profiles, Figure 1b, it results in two peaks related to elastically-strained and relaxed portions of the sample. Using lattice parameters of *a*_GaAs_ = 0.5653 nm and *a*_GaSb_ = 0.6096 nm, the chemical composition index was evaluated as *y* = 0.022 under the assumption of Vegard’s rule. The experimentally evaluated chemical composition is reasonably close to the nominal value of 0.03, which we will use in most chemical formulas for simplicity.

In contrast to the Al-free samples, the XRD profiles of the LT-Al_1−*x*_Ga*_x_*As and LT-Al_1−*x*_Ga*_x_*As_1−*y*_Sb*_y_* samples always demonstrate two well-separated peaks related to the substrate and the epitaxial film. The position of the film-related 004 reflection is almost not affected by the annealing indicating that the lattice parameter of the film is not changed. It is due to the intermediate anneals during the epitaxy described above. In fact, among the whole film, only its final portion remained unannealed, which is as thin as 160 nm and 100 nm for the LT-Al*_1−x_*Ga*_x_*As and LT-Al_1−*x*_Ga*_x_*As_1−*y*_Sb*_y_* samples, correspondingly. These parts make only a weak contribution to the entire XRD patterns. The corresponding changes are too small to be reliably extracted for calculations of the [As_Ga_] values with a reasonable accuracy.

The angular positions of the two well-separated XRD peaks are used for a precise evaluation of the aluminum concentration *x* in the Al_1−*x*_Ga*_x_*As film assuming the Vegard’s rule and taking the lattice parameter of unstrained AlAs equal to *a*_AlAs_ = 0.5662 nm. The derived value is *x* = 0.602. This value is very well consistent with the nominal quantity of 60% utilized in the chemical formulas within this paper.

In the LT-Al_1−*x*_Ga*_x_*As_1−*y*_Sb*_y_* sample, the lattice expansion of the film compared to the GaAs substrate is a result of the contamination of Al in the cation sublattice and Sb in the anion sublattice. The two contributions cannot be separated by the XRD study solely. It will be done below by a joint analysis of the XRD and optical data in Section 3.3.

### 3.2. Transmission Electron Microscopy

**Figure 2:**
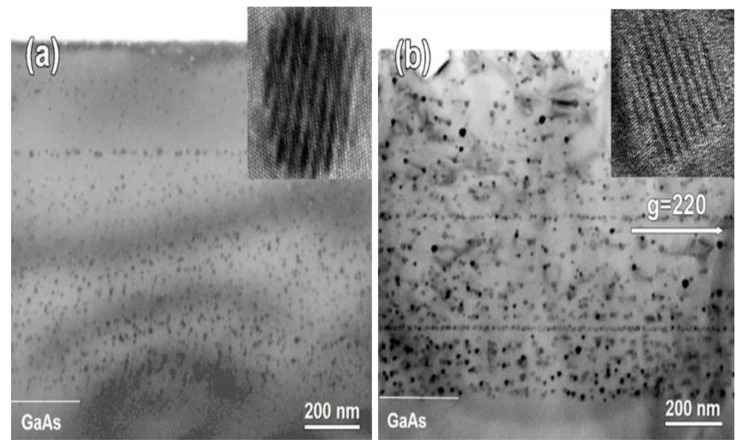
Bright-field cross-sectional TEM micrographs of the samples annealed at 600 °C: (**a**) LT-GaAs ([110] zone axes); (b) LT-GaAs0.97Sb0.03 (two-beam conditions, g = 220). The insets are high-resolution images of individual nanoinclusions.

Examples of cross-sectional TEM images showing an overview of the LT-GaAs and LT-GaAs_0.97_Sb_0.03_ layers annealed at 600 °C are presented in Figure 2. The actual thicknesses of the LT-GaAs and LT-GaAs_0.97_Sb_0.03_ layers deduced from the TEM images are 940 nm and 880 nm, correspondingly.

The micrographs in Figure 2 visualize the nanoinclusions as generally disordered arrays of small rounded dark spots. Insertions show single particles in high-resolution mode containing moire fringes, which testify to the crystalline nature of the inclusions. The growth interruptions with in-situ low-temperature anneals, which were performed for smoothing the growth surface, are also revealed as horizontal lines of the dark spots. These lines are projections of quasi-2D arrays of the nanoparticles decorating the interfaces formed by the growth interruptions and anneals.

The size distribution of the As nanoparticles in GaAs is rather wide as a result of the stochastic self-organization process [15]. The mean diameter of the nanoparticles is evaluated as *D_p_* = 9.3 nm, whereas the largest observed nanoparticle has a diameter of 22 nm. It is evident from Figure 2a that the As-GaAs metamaterial is free of dislocations and other extended defects. The As nanoinclusions in GaAs are known to have a rhombohedral crystal structure of A7 type, inherent to bulk As and Sb under normal ambient conditions [20,21]. The atomic volume of As in the A7 structure is very close to the atomic volume in the zinc-blende GaAs lattice. Therefore, no local stresses are induced by the formation of the As nanoinclusions in the GaAs matrix. Consequently, there is no substantial driving force for the defect formation in the As-GaAs metamaterial.

The situation is different in the case of LT-GaAs_0.97_Sb_0.03_. The micrograph in Figure 2b reveals dislocation loops adjacent to most of the nanoparticles. Such dislocation loops were previously found in LT-GaAsSb and identified to be prismatic with the Burgers vector directed along the <001> matrix axis [22,23]. Their generation is associated with the particle–matrix lattice mismatch due to the incorporation of antimony into nanoinclusions. The threshold nanoinclusion diameter for the dislocation loop formation is about 8 nm. In our case, the mean particle size is measured to be about 12 nm, which substantially exceeds the threshold.

**Figure 3:**
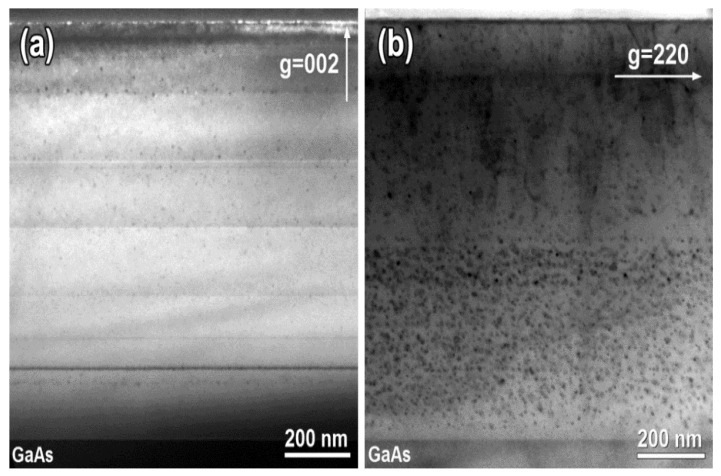
Cross-sectional TEM micrographs of (**a**) LT-Al_0.6_Ga_0.4_As (002 dark-field); (**b**) LT-Al_0.6_Ga_0.4_As_0.97_Sb_0.03_ (220 bright-field) the samples annealed at 600 °C.

Figure 3 depicts an overview of the LT-Al_0.6_Ga_0.4_As and LT-Al_0.6_Ga_0.4_As_0.97_Sb_0.03_ layers after annealing at 600 °C. The fine contrast lines separating the areas of continuous growth are well pronounced in the image of the LT-Al_0.6_Ga_0.4_As layer (Figure 3a) grown with six growth interruptions. The total thickness of the LT-Al_0.6_Ga_0.4_As layer is 960 nm. The nanoinclusions are inhomogeneously dispersed over the layer thickness with their highest density in the area adjacent to the substrate. The mean diameter of the As nanoparticles in the Al_0.6_Ga_0.4_As layer is evaluated as *D_p_* = 6.0 nm. No extended defects are revealed by the TEM investigations.

As follows from the TEM images, of which an example is shown in Figure 3b, the total thickness of the LT-Al_0.6_Ga_0.4_As_0.97_Sb_0.03_ layer is 940 nm. A developed array of extended defects is formed in the upper part of the layer. A dense ensemble of nanoinclusions is seen in the lower 450 nm thick part of the layer. Some particles are found to have adjacent dislocation loops similar to those in the LT-GaAs_0.97_Sb_0.03_ sample (Figure 3b). While a mean particle diameter of 6 nm is smaller than the threshold size for the dislocation loop formation, a wide size dispersion of the nanoinclusions in the Al_0.6_Ga_0.4_As_0.97_Sb_0.03_ layer provides a substantial amount of the nanoparticles with diameters from 8 to 14 nm, which are bigger than the threshold size for the dislocation loop formation. We note again that there are no dislocation loops in the Sb-free LT-Al_0.6_Ga_0.4_As sample.

**Figure 4:**
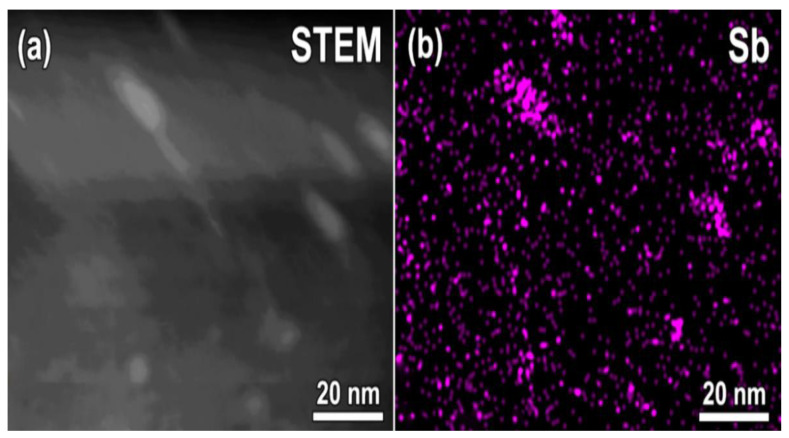
LT-Al_0.6_Ga_0.4_As_0.97_Sb_0.03_ layer annealed at 600 °C: (**a**) Transmission electron micrograph in the STEM-HAADF mode; (**b**) Sb distribution map of the same area collected by EDXS.

While Al, Ga, and As being the epitaxial layer constituents are uniformly distributed, the accumulation of antimony in precipitates should be perceptible by EDXS against the background of its low content in the matrix. The nano-inclusions in the LT-Al_0.6_Ga_0.4_As_0.97_Sb_0.03_ layer appear to contain a high concentration of Sb, as evidenced by the STEM-HAADF images and by the local analysis of the elemental composition by EDXS. Figure 4a presents a STEM-HAADF image of a small area in Al_0.6_Ga_0.4_As_0.97_Sb_0.03_ sample, where the nanoinclusions are seen as bright spots due to increased scattering of the transmitted electrons. The EDXS map of the Sb distribution collected over the same area using Sb *L_α_*-line is shown in Figure 4b. As can be seen from a comparison of both images, the enhanced scattering of electrons on the nanoinclusions is due to a high content of the heavy antimony atoms in them, that is, nanoinclusions are composed of an AsSb alloy.

**Figure 5:**
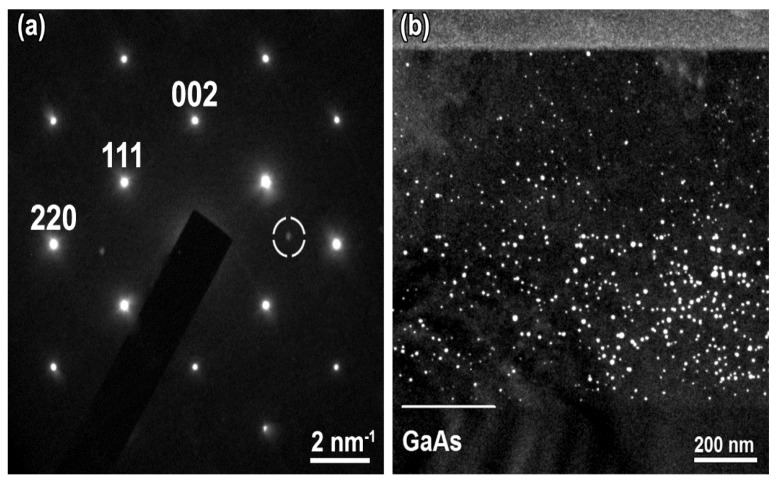
(**a**) Electron diffraction pattern and (**b**) micrograph acquired using the reflex of the second phase marked with dashed circle in panel (**a**). The data are for the layer of LT-Al_0.6_Ga_0.4_As_0.97_Sb_0.03_ annealed at 600 °C.

To estimate the concentration of the AsSb nanoparticles, we use imaging in a weak reflection from the AsSb nanoinclusions appearing in the diffraction pattern. Figure 5a shows such a diffraction pattern with strong reflections from the Al_0.6_Ga_0.4_As_0.97_Sb_0.03_ cubic zinc-blend matrix and two weak spots located approximately along the [110] matrix direction. These spots are due to the second phase represented by the nanoinclusions. The presence of only two reflections of the second phase does not allow us to identify the microstructure of the nanoparticles. However, the image in Figure 5b recorded under two-beam diffraction conditions using the second phase reflection circled in Figure 5a clearly visualizes the nanoparticle distribution over the layer thickness. As can be seen, the nanoinclusions are formed in all portions of the layer, however, their concentration is much higher in the lower part than in the upper portion.

Processing of the TEM images recorded under various diffraction conditions makes it possible to determine the mean diameter *D_p_* and concentration *N_p_* of nanoinclusions. The volume fraction, which the array of nanoinclusions occupy in the matrix *f*, is found using the particle concentration *N_p_* and mean particle volume *V_p_* determined as an average of the volumes of individual particles. The results are presented in Table 1.

The obtained data on the mean particle volume *V_p_* and their concentration *N_p_* are used to evaluate the concentration of antisite defects [As_Ga_]_(TEM)_ in the as-grown samples. The concentration of atoms contained in the nanoinclusions *N_atom_* can be determined using the unit cell volume *V_cell_* and the number of atoms *Z* in it as follows

(2) $$N_{atom}=\frac{V_{p}}{V_{cell}}\;\cdot \;Z\;\cdot \;N_{p}$$

The rhombohedral unit cell of type A7 contains two atoms, and its volume for As is 0.044907 nm^3^. For LT-GaAs_0.97_Sb_0.03_ and LT-Al_0.6_Ga_0.4_As_0.97_Sb_0.03_, a high concentration of Sb in the nanoinclusions should be taken into account. The volume of the rhombohedral Sb unit cell is 0.064267 nm^3^. As follows from the ratio of the intensities of the Ga, As, and Sb peaks in the EDXS spectra recorded outside and within the region of the inclusion (not shown here), the antimony concentration inside the inclusions is very roughly estimated as 50%. Accordingly, the unit cell volume averaged between As and Sb was used in the calculations. Taking into account that the inclusions are formed equally by the atoms of the anion sublattice and anti-site defects As_Ga_ and Sb_Ga_ in the cation sublattice, the concentration of antisite defects in the as-grown samples should be half the concentration of atoms contained in inclusions after annealing.

The concentration of antisite defects [As_Ga_]_(TEM)_ obtained for LT-GaAs and LT-GaAs_0.97_Sb_0.03_ turned out to be the same within the measurement error and equal to 1.1 × 10^20^ cm^−3^. For LT-Al_0.6_Ga_0.4_As and LT-Al_0.6_Ga_0.4_As_0.97_Sb_0.03_, it was markedly lower, 6.5 × 10^19^ cm^−3^ and 7.2 × 10^19^ correspondingly.

### 3.3. Optical Study

The experimental optical extinction coefficient spectra are determined using the Beer–Lambert law

(3) $$\alpha =-\ln\left(T_{\alpha }/T_{0}\right)/d,$$

where $T_{\alpha }$ is the experimental optical transmission spectra of the samples, $T_{0}$ is the reference optical transmission spectrum of stoichiometric GaAs grown under conventional temperature conditions with a very low concentration of optically-active defects and impurities. Our XRD examination shows that the intermediate annealing provides almost complete relaxation of the excess-arsenic-related lattice expansion, which means the transformation of the optically-active As_Ga_ antisite defects into the array of nanoinclusions revealed by TEM. Therefore, in the calculations of the extinction coefficient related to the As_Ga_ antisite defects in the as-grown samples, we use the actual thicknesses, *d*, of the unannealed portions of the epilayer. The corresponding values are 0.29 μm and 0.42 μm, respectively, for the LT-GaAs and LT-GaAs_0.97_Sb_0.03_ films.

**Figure 6:**
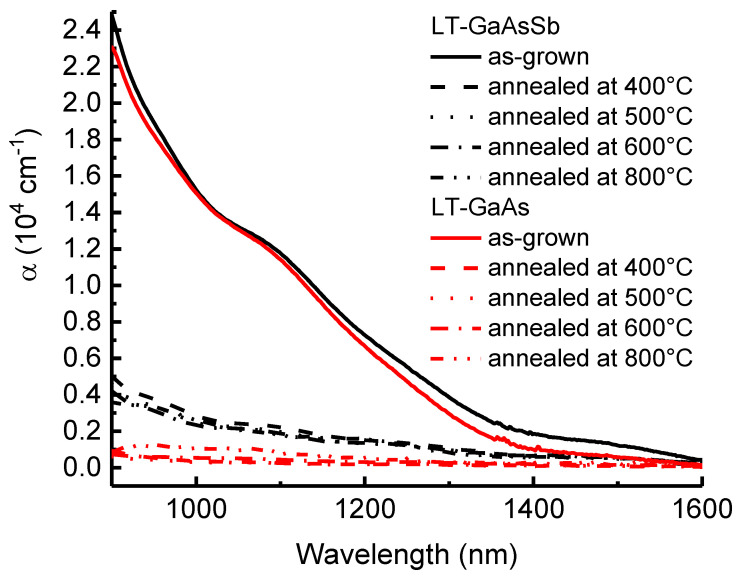
Spectra of the optical extinction coefficient for the LT-GaAs and LT-GaAs_0.97_Sb_0.03_ samples before and after annealing at different temperatures.

Figure 6 shows the optical extinction spectra for the LT-GaAs and LT-GaAs_0.97_Sb_0.03_ samples before and after annealing at different temperatures. The as-grown parts of the samples are characterized by broad tails of light absorption up to a wavelength of 1400 nm. These spectral tails are known to originate from the absorption of light by the system of As_Ga_ antisite defects in the low-temperature GaAs epilayer [5,8]. Concentrations of nonstoichiometric As_Ga_ centers determined via the calibration [19] are $1.2\times 10^{20}\;{\mathrm{cm}}^{-3}$ for both the LT-GaAs and LT-GaAs_0.97_Sb_0.03_ samples.

The values obtained for the molar fractions *x* and *y* of the components and the concentration of antisite defects [As_Ga_] by different measurement techniques are summarized in Table 2.

As seen from Table 2 the values deduced from the optical extinction spectra are in good agreement with the corresponding data obtained by our TEM investigations and they are in satisfactory agreement with the X-ray diffraction data.

The post-growth annealing leads to a strong decrease in the As_Ga_-related optical absorption for both the LT-GaAs and LT-GaAs_0.97_Sb_0.03_ samples, as demonstrated in Figure 6. It is a result of the precipitation of the point defects into arrays of crystalline nanoinclusions when the diffusion of defects is thermally activated. This phenomenon is also accompanied by the decrease in the lattice parameter of the semiconductor matrix revealed by the XRD study (Figure 1) and by the appearance of nanoinclusion contrasts in the TEM images (Figure 2). So, the phase transformation is revealed and monitored by the three independent experimental techniques.

Since the light extinction based on the point defects is eliminated after the post-growth annealing, the absorption and scattering of light in the annealed samples can be caused by the self-organized system of As or AsSb nanoinclusions [6,7,8,10,11,12,13]. In the Sb-free LT MBE epilayers, the nanoinclusions are composed of arsenic atoms only. It is evident from Figure 6 that the optical extinction in the region of 900–1400 nm is rather weak in this case. This observation agrees with previous reports [6,7,8]. In the antimony-containing low-temperature-grown solid solutions, the nanoparticles are strongly enriched with antimony [24,25], which is confirmed by the comparison of the STEM and EDXS images in Figure 4. It is evident from Figure 6 that the enrichment of the nanoinclusions with Sb atoms leads to a notable optical extinction tail, which is almost independent of the annealing conditions in a very wide range. This observation agrees with previous reports [12,13].

**Figure 7:**
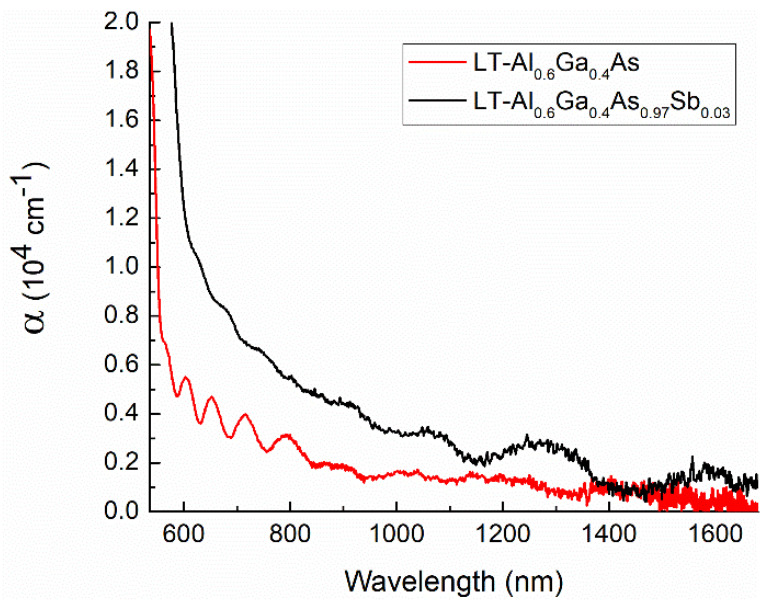
Optical extinction spectra of LT-Al_0.6_Ga_0.4_As (black) and LT-Al_0.6_Ga_0.4_As_0.97_Sb_0.03_ (red) films annealed at 600 °C for 15 min.

The experimental spectra of the light extinction coefficient for the LT-Al_0.6_Ga_0.4_As and LT-Al_0.6_Ga_0.4_As_0.97_Sb_0.03_ samples annealed at 600 °C are shown in Figure 7. The spectra are very similar to those of the samples annealed at other temperatures. For these measurements, the GaAs substrate was removed, and the films were transferred to an optical glass. A strong reflection from the air-semiconductor interfaces results in a pronounced Fabry–Perrot pattern in the extinction spectra.

The optical extinction increases strongly for light with a short wavelength, which corresponds to the absorption in the semiconductor matrix. Both Al_0.6_Ga_0.4_As and Al_0.6_Ga_0.4_As_0.97_Sb_0.03_ have indirect band gaps, however, the direct band-to-band transitions at the Γ-point of the Brillouin zone are slightly above. These transitions manifest themselves by an abrupt increase in the absorption coefficient, whereas the absorption due to indirect transitions is relatively weak and thresholdless. The onset of the optical absorption related to the direct band gap transitions is different for the Al_0.6_Ga_0.4_As and Al_0.6_Ga_0.4_As_0.97_Sb_0.03_ samples due to the presence of antimony in the latter case, as well as due to a possible variation of the aluminum content. The data on the band gap shift along with the XRD data on the lattice parameter of the alloy allows us to separately evaluate the *x* and *y* parameters in the chemical formula of Al_x_Ga_1−x_As_1−y_Sb_y_. For that we solve the following set of equations [25]:

(4) $$a_{Al_{x}Ga_{1-x}As_{y}Sb_{1-y}}=xya_{AlSb}+x\left(1-y\right)a_{AlAs}+\left(1-x\right)ya_{GaSb}+\left(1-x\right)\left(1-y\right)a_{GaAs}\;$$

(5) $$\Delta E_{\Gamma }\left(x,y\right)=1.155x+0.37x^{2}-1.9y+1.2y^{2}-0.839,$$

where (4) represents Vegard’s rule for the lattice parameter. The Equation (5) in the set defines a shift of the direct band gap of Al*_x_*Ga_1−*x*_As_1−*y*_Sb*_y_* compared to the Al_0.608_Ga_0.392_As reference measured in eV [26,27]. The derived values are x=0.553 and y=0.037. For this chemical composition, the direct band gap $E_{\Gamma }$ is only 10–15 meV above the indirect $E_{L}$.

A distinctive feature of the optical extinction spectra of Al_0.6_Ga_0.4_As_0.97_Sb_0.03_ in Figure 7 is a wide extinction band, which gradually diminishes in the range from 600 nm to about 1200 nm. Similar, but the weaker band is also characteristic of Al_0.6_Ga_0.4_As. In this case, the substantial absorption disappears for a wavelength of 900 nm and above. This absorption cannot be due to indirect electronic transitions in the matrix. Also, it cannot be due to the of As_Ga_ antisite defects, since the samples are annealed at high temperatures. In accordance with the XRD data, the excess-arsenic-related lattice distortion is completely relaxed. The developed arrays of the AsSb nanoinclusions, which should be responsible for the extinction [12,13], are visualized by TEM. In the annealed LT-GaAs_0.97_Sb_0.03_ sample, the extinction grows up to 0.4 × 10^4^ cm^−1^ with increasing photon energy (decreasing wavelength of light) till the semiconductor matrix becomes opaque due to the band-to-band optical absorption. In the case of the Al_0.6_Ga_0.4_As_0.97_Sb_0.03_ sample, the band gap is wider, and the extinction coefficient keeps growing up to a value of 1 × 10^4^ cm^−3^. The optical properties of the AsSb-Al_0.6_Ga_0.4_As_0.97_Sb_0.03_ and AsSb-GaAs_0.97_Sb_0.03_ metamaterials appear to be almost independent of the post-growth annealing temperature, which was varied from 400 to 800 °C in this study. For the AsSb-GaAs_0.97_Sb_0.03_ sample, this fact can be monitored in Figure 6. For the system of As nanoparticles the corresponding absorption is substantially weaker (Figure 7) since the plasmon resonance occurs at higher energy where the Al_0.6_Ga_0.4_As matrix is opaque.

## 4. Discussion

The in situ data acquired during the LT MBE and ex situ data provided by structural characterization of the LT-Al_0.6_Ga_0.4_As and LT-Al_0.6_Ga_0.4_As_0.97_Sb_0.03_ epitaxial films reveal substantial problems arising from the high Al concentration in the solid solutions. Compared correspondingly to the LT-GaAs and LT-GaAs_0.97_Sb_0.03_ epitaxial films grown under the same conditions, the films with the 60% Al concentration exhibit much lower critical thickness, below which the planarity of the growth surface is maintained, and high crystalline quality of the growing film is provided. This critical thickness appears also substantially lower than those for LT-Al_0.3_Ga_0.7_As and LT-Al_0.3_Ga_0.7_As_0.97_Sb_0.03_ studied previously [13,25].

The morphological instability is governed by the mismatch strain in the growing film combined with the surface energy and diffusion of adatoms. The lattice mismatch between the GaAs substrate and the film is raised with Al and Sb concentrations. It is additionally enhanced during the low-temperature epitaxy due to the incorporation of the As antisite defects, as shown in the XRD section above. Thus, the solid solutions with the high Al concentration provide a stronger thermodynamic driving force for the development of surface relief. The formation of the surface relief is mediated by the diffusion of adatoms. The latter is associated with thermal activation over the diffusion barriers. It is known that the diffusion barrier for Al adatoms on the (001) GaAs surface is higher than for Ga adatoms [28]. Therefore, the films with high Al concentration are much more sensitive to the reduction of the growth temperature.

Despite the above problems, we have succeeded to grow rather perfect LT-Al_0.6_Ga_0.4_As and LT-Al_0.6_Ga_0.4_As_0.97_Sb_0.03_ layers by repeated intermediate anneal during the MBE process. This technological procedure leads, on the one hand, to partial relaxation of the mismatch strain due to the phase transformations in the film bulk, and, on the other hand, it flattens the growth surface due to the enhanced migration of the adatoms.

Another problem arises when the post-growth annealing is applied to form the As or AsSb nanoinclusions in the LT-Al_0.6_Ga_0.4_As or LT-Al_0.6_Ga_0.4_As_0.97_Sb_0.03_ layers. It is known that the diffusion of the excess As in LT-AlAs is inhibited [29]. This phenomenon can be used, for instance, to prevent an intermixing between LT-GaAs layers with different excess As concentrations [15]. The diffusion of the excess As in the bulk seems to slow with increasing Al content from *x* = 0 (i.e., in LT-GaAs) to *x* = 0.3 (see Ref. [13]) and to *x* = 0.6. In fact, after annealing at 600 °C, the mean particle size in the LT-Al_0.6_Ga_0.4_As_0.97_Sb_0.03_ is 6.0 nm, whereas after the same annealing, the mean particle size in the LT-Al_0.3_Ga_0.7_As_0.97_Sb_0.03_ is 7.5 nm [13], and it is 11.9 nm in the LT-GaAs_0.97_Sb_0.03_ (see Table 1). In addition, after annealing at 600 °C, the mean particle size in the LT-Al_0.6_Ga_0.4_As is 6.0 nm, whereas in the LT-GaAs it is 9.3 nm (Table 1). Fortunately, the retardation of the self-organization processes in the LT-Al_0.6_Ga_0.4_As or LT-Al_0.6_Ga_0.4_As_0.97_Sb_0.03_ layers does not appear to be critical. In both samples, we successfully formed developed systems of the nanoinclusions, which accumulate almost the whole thermodynamically allowed amount of the super-stoichiometric atoms of group V. The volume fraction occupied by the nanoinclusions in the composite metamaterial, according to the TEM data, is *f* = 0.3–0.4%. This value is reasonably well consistent with the XRD data and with the data from our optical investigations of both absorption by the As_Ga_ antisite defects and by the plasmonic AsSb nanoparticles.

Previous investigations [22,23] established As inclusions in LT-GaAs to have the rhombohedral crystal structure of A7 type, inherent to As and Sb under normal ambient conditions. It is often described in the hexagonal axes. The particle–matrix orientation relationship for the As nanoinclusions was determined to be ${\left[0001\right]}_{p}∥111_{m}$ and ${\left\{0\bar{1}14\right\}}_{p}∥{\left\{110\right\}}_{m}$, where the indices *p* and *m* correspond to the particle and matrix, respectively. In the case of Ga(As,Sb) and (Al,Ga)(As,Sb) solid solutions, the incorporation of Sb into the nanoinclusions may result in a modification of the particle–matrix orientation relationship and even in a rearrangement of the particle microstructure due to the strengthened particle–matrix mismatch strain. Indeed, the mismatch strain results in the generation of dislocation loops connected to the AsSb nanoinclusions, which are larger than 8 nm in diameter [20,21], as can be seen in Figure 2b. Such dislocation loops have never been observed for the As nanoparticles in GaAs and AlGaAs (Figure 2a and Figure 3a). Moreover, significant changes in the particle appearance and electron diffraction pattern have been observed for the AsSb nanoinclusions by TEM after annealing at 600 °C [24,30]. The AsSb inclusions take the ordinary microstructure and particle–matrix orientation relationship when the annealing temperature is elevated to 750 °C [25].

So, the structural investigations have documented the formation of developed systems of As nanoinclusions in the bulk of GaAs and Al_0.6_Ga_0.4_As epitaxial films and AsSb nanoinclusions in the bulk of GaAs_0.97_Sb_0.03_ and Al_0.6_Ga_0.4_As_0.97_Sb_0.03_ epitaxial films. The As or AsSb nanoinclusions occupy from 0.3 to 0.6% of the total volume of the metamaterial.

The resulting Al_0.6_Ga_0.4_As_0.97_Sb_0.03_ metamaterial exhibits a strong excess optical extinction near its bandgap. Substantial tails in the optical extinction were previously observed in annealed samples of Al_0.3_Ga_0.7_As_0.97_Sb_0.03_, where a similar system of the AsSb nanoinclusions was formed [12,13]. These tails were attributed to the Fröhlich plasmon resonance. The experimental optical data in Refs. [12,13] recorded in the transparency window of the Al_0.3_Ga_0.7_As_0.97_Sb_0.03_ matrix are well-consistent with the plot in Figure 7. It is important that due to higher aluminum content the optical window covered in this paper is substantially wider. It allows us to monitor the plasmon-related optical absorption in the photon energy range from 1 eV to 2 eV. A detailed physical analysis of this absorption is beyond the scope of this paper. However, it is worth noting that the plasmon absorption can provide an ultrashort relaxation time of about 1 fs with an absorption coefficient as high as 10^4^ cm^−1^. The 1 μm thick films under study are quite efficient optical absorbers near 700 nm. The implementation of the intermediate anneals makes it possible to produce thicker films of the AsSb-AlGaAsSb nanomaterials. It is important that the fabrication technology of the AsSb-AlGaAsSb nanomaterials is fully compatible with the common technology of MBE, which is widely utilized in the industrial production of semiconductor optoelectronic devices. Then, the layers with a built-in system of the plasmonic AsSb nanoinclusions can be integrated into optoelectronic and photonic chips within a single process of MBE.

## 5. Conclusions

Our investigations show that epitaxial films of Al_0.6_Ga_0.4_As and Al_0.6_Ga_0.4_As_0.97_Sb_0.03_ solid solutions can be grown with a high excess of group-V elements by the low-temperature MBE at a temperature as low as 200 °C at least 1 µm thick. A combination of the anneals during the epitaxy and after it allows us to form a developed system of As or AsSb nanoinclusions, which occupy 0.3–0.6% of the material volume. While the As nanoinclusions formed in Al_0.6_Ga_0.4_As are optically inactive, the AsSb nanoinclusions self-organized in Al_0.6_Ga_0.4_As_0.97_Sb_0.03_ provide a strong optical absorption near the band edge of the semiconductor matrix due to the Fröhlich plasmon resonance. Owing to the wider bandgap of the grown Al_0.6_Ga_0.4_As_0.97_Sb_0.03_ compound, we have expanded the spectral range available for studying the Fröhlich plasmon resonance. Such a material represents an optically active medium of which the formation process is completely compatible with the epitaxial growth technology of semiconductors.

## Figures and Tables

**Table 1:** Parameters of the nanoparticles in the samples annealed at 600° according to TEM.

Sample	Mean Particle Size, *D_p_*, nm	Concentration in the Matrix, *N_p_,* cm^−3^	Mean Particle Volume, *V_p_*, cm^3^	Volume Fraction in the Matrix, *f*
LT-GaAs	9.3	5.9 × 10^15^	8.7 × 10^−19^	5.1 × 10^−3^
LT-GaAs_0.97_Sb_0.03_	11.9	4.2 × 10^15^	1.4 × 10^−18^	5.9 × 10^−3^
LT-Al_0.6_Ga_0.4_As	6.0	2.1 × 10^16^	1.4 × 10^−19^	2.9 × 10^−3^
LT-Al_0.6_Ga_0.4_As_0.97_Sb_0.03_	6.0	2.0 × 10^16^	2.0 × 10^−19^	3.9 × 10^−3^

**Table 2:** Chemical composition of the samples and concentration of the antisite defects.

Sample	*x*	*y*	[As_Ga_]_(XRD),_ cm^−3^	[As_Ga_]_(optics),_ cm^−3^	[As_Ga_]_(TEM),_ cm^−3^
LT-GaAs	0	0	4.8 × 10^19^	1.2 × 10^20^	1.1 × 10^20^
LT-GaAs_0.97_Sb_0.03_	0	0.022	7.5 × 10^19^	1.2 × 10^20^	1.1 × 10^20^
LT-Al_0.6_Ga_0.4_As	0.602	0	-	-	6.5 × 10^19^
LT-Al_0.6_Ga_0.4_As_0.97_Sb_0.03_	0.553	0.037	-	-	7.2 × 10^19^
